# Energy dispersive x-ray spectroscopy for nanostructured thin film density evaluation

**DOI:** 10.1088/1468-6996/16/2/025007

**Published:** 2015-04-08

**Authors:** Irene Prencipe, David Dellasega, Alessandro Zani, Daniele Rizzo, Matteo Passoni

**Affiliations:** Dipartimento di Energia, Politecnico di Milano, Milan, Italy

**Keywords:** density evaluation, thin film, EDS, foam, areal density, 68.37.Hk, 78.70.En

## Abstract

In this paper, we report on two fast and non-destructive methods for nanostructured film density evaluation based on a combination of energy dispersive x-ray spectroscopy for areal density measurement and scanning electron microscopy (SEM) for thickness evaluation. These techniques have been applied to films with density ranging from the density of a solid down to a few 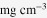, with different compositions and morphologies. The high resolution of an electron microprobe has been exploited to characterize non-uniform films both at the macroscopic scale and at the microscopic scale.

## 1. Introduction

In recent decades, the production and characterization of thin films with a wide range of morphologies and compositions have attracted great interest due to their applicative potential. Therefore, appropriate characterization methods are required for film properties and, in particular, for density, which is a key parameter for many applications, among which laser-plasma interaction and laser driven ion acceleration experiments have recently triggered wide interest [1, 2].

An ideal technique for thin film density measurement should be reliable in a wide density range (from the density of a solid to a few 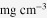) and for a great variety of materials and morphologies. It should allow us to evaluate the density of non-homogeneous films with a good spatial resolution. It should also be non-destructive and fast and require a simple and cheap experimental apparatus.

In general, the density of thin films can be evaluated by combining thickness and areal density measurements. The former can be achieved, for example, through electron microscopy, by analysing cross-sectional scanning electron microscopy (SEM) images or by transmission electron microscopy (TEM), depending on the order of magnitude. Several methods can be employed to measure areal density. Commonly used nuclear-based techniques such as Rutherford backscattering spectroscopy (RBS), elastic recoil detection analysis, and nuclear reaction analysis provide accurate areal density measurements with a good spatial resolution, but they require complex experimental equipment, i.e., linear accelerators to produce MeV ion beams. Conversely, in thin film deposition facilities, a quartz crystal microbalance (QCM) is often adopted to measure the mass deposition rate on a well-defined surface and, therefore, a mean areal density value. This technique is very popular due to the simplicity of the required instrument. However, QCM only allows us to measure the average areal density of a film directly deposited on its quartz crystal surface in conditions simulating the growth configuration of the film under analysis, thus providing an indirect measurement. Moreover, this method is not reliable for very low density materials (below 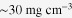), as shown for carbon foams in [3].

In this context, an attractive technique satisfying most of the ideal requirements listed above is based on energy dispersive x-ray spectroscopy (EDS). The energy and intensity of characteristic x-rays produced in matter by an incident electron beam are related to the atomic number of the emitting element and areal density of the examined layer, respectively. The penetration depth of electrons in matter is a function of the electron accelerating voltage and ranges approximately from 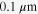 to several 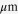 for standard electron probe beams (2–50 keV). As a consequence, a proper selection of the electron accelerating voltage allows us to characterize a surface layer of the sample under investigation, i.e., a thin coating deposited on the sample surface. In the 1960s, Sweeney *et al* proposed to employ EDS for the evaluation of the thickness of compact films with known density [4]. Nevertheless, this technique has never been used for density evaluation. An EDS-based method would be non-destructive and could provide local density values, allowing us to characterize non-homogeneous films. Moreover, the microanalysis equipment required for EDS is relatively simple, and it is often integrated into SEM devices, which are commonly used in material science laboratories and allow us also to achieve thickness measurements.

In this paper, we quantitatively develop and test two methods for thin film density evaluation, both based on the combined use of EDS for areal density measurement and cross-sectional SEM images for thickness assessment. The main goal of this work is to show the applicability of these methods and to study their limits. To this purpose, they have been employed to characterize compact coatings and nanostructured thin films with various compositions, a large variety of mesoscale morphologies, and density ranging from the density of a solid to a few 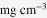. Thermal evaporation and pulsed laser deposition (PLD) have been exploited for film growth, as described in section 3. To better illustrate the methods employed for areal density evaluation, we will give an account of a few EDS-based methods for thickness evaluation in section 2.1. In section 2.2, we will describe the most relevant theoretical aspects available in the literature about areal density evaluation as well as a few practical aspects concerning the experimental setup. In particular, criteria for the choice of a measurement method and for the appropriate selection of the electron accelerating voltage will be discussed. Experimental results will be illustrated and discussed in section 4.

## 2. EDS-based methods for thin film areal density evaluation

### 2.1. General background

The application of EDS to coating thickness evaluation has been widely explored since the 1960s. To this aim, a number of methods have been employed in the literature. For example, thickness evaluation was achieved by measuring the minimum accelerating voltage required to probe the whole film thickness [5] or the accelerating voltage, for which a given fraction of the x-ray intensity produced by a reference standard is emitted by the sample [6]. Here, we discuss the application of two methods proposed by Sweeney, Seebold, and Birks in 1960 [4] and by Cockett and Davis in 1963 [7], respectively, because of their relevance to this work.

**Figure 1.**
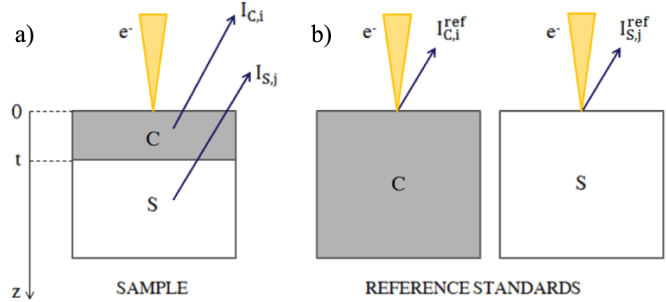
EDS-based film thickness measurement methods. a) X-ray emission from a coating with thickness *t* and from a substrate due to the incident electron beam. b) X-ray emission from reference standards for the coating method and the substrate method.

These approaches, respectively known as the *coating method* and the *substrate method*, were developed for multilayer samples composed of a known substrate and a coating with unknown thickness. In these methods, the coating layer thickness is calculated from the intensity of x-rays produced either in the sample coating or substrate by an incoming electron beam with appropriate initial energy, provided that the intensity of the x-rays produced by a bulk reference standard is known (see figure 1). The main difference between the coating method and the substrate method lies in the choice of the reference standard: the reference standard must contain an emitting element present only in the sample coating or in the substrate, respectively.

The reliability of the EDS-based methods described so far for thickness evaluation was thoroughly investigated in the literature. Thickness values measured by employing EDS-based methods were compared to values achieved using other techniques, i.e., RBS or cross-sectional SEM images. The difference between results achieved with established techniques and with EDS-based methods is generally around 15–20% [7]. Although these methods were proposed for thickness evaluation, the parameter they are directly sensitive to is the areal density, since the generation of characteristic x-rays in a layer does not depend on the material thickness *t* or density *ρ* separately, but on its areal density, 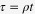. Therefore, this technique can be applied to density evaluation for thin films with known thickness. In the next subsection, we will illustrate theoretical aspects related to areal density evaluation and x-ray production modelling, and we discuss issues related to the experimental setup.

### 2.2. Areal density evaluation

The calculation of film areal density from x-ray intensity requires knowledge of the so-called probability function for x-ray production (PFXP), 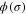. This function, introduced by Castaing in 1951 [8], describes the distribution in depth of the primary ionizations produced in a sample by an incoming electron. The function argument is a depth expressed in terms of areal density, and is given by 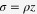, where *z* is the depth measured in linear units.

PFXP allows us to calculate the x-ray intensity 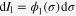 emitted by an element *Z*_l_ in a layer 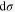 at a depth *z* below the sample surface of a material with density *ρ*. Thus, the intensity measured for a characteristic x-ray line emitted by *Z*_l_ in a layer with finite areal density 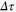 is1
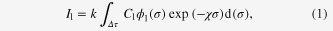
where *C*_l_ is the mass concentration of the element under analysis. The term 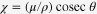 takes into account the absorption of emitted x-rays propagating to the sample surface: 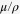 is the mass absorption coefficient, and *θ* is the x-ray take-off angle. *k* is a constant given by2
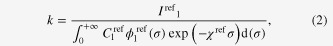
where 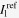 is the x-ray intensity produced by *Z*_l_ present in a reference standard with known composition. The ref superscript indicates that the concentration of *Z*_l_, the PFXP, and the absorption term *χ* refer to the reference standard. X-rays intensities must be measured under the very same conditions for the sample under analysis and the reference standard, since the intensity of detected characteristic x-rays is influenced by many factors, such as the beam current, the measurement duration, and geometry.

If a model for PFXP evaluation is available, the relation between the unknown coating areal density, *τ*, and the x-ray intensity emitted by the sample can be calculated through equations (1) and (2) for both methods.

In the coating method, characteristic x-rays emitted by an element *Z*_i_ present in the sample coating are considered. The intensity of the selected x-ray line is calculated for both sample and reference standard from the recorded spectra by integrating the peak fitting curves. According to equations (1) and (2), the coating to reference standard intensity ratio 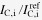 can be expressed in terms of film areal density through the following formula3
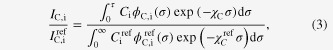
where the coefficients 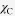 and 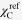 account for the absorption of x-rays in the sample coating and in the reference standard, respectively.

In the substrate method, the intensity of a characteristic x-ray line emitted by an element *Z*_j_ contained in the sample substrate is considered. The sample substrate to reference standard x-ray intensity ratio 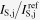 can be expressed as a function of film areal density *τ* through equations (1) and (2). If x-ray absorption in the coating is taken into account, the relation between the intensity ratio and *τ* can be expressed as follows4
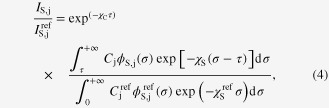
where the coefficients 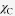, 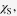 and 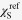 introduce the effect of x-ray absorption in the sample coating and substrate and in the reference standard.

Once the x-ray intensities emitted from the sample and from an appropriate reference standard have been measured, areal density *τ* can be calculated by inversion of equation (3) for the coating method or (4) for the substrate method. Nevertheless, repeating this process for each set of experimental data can be time consuming. Thus, it is convenient to calculate calibration curves expressing areal density as a function of the sample to a standard intensity ratio for a given substrate–coating combination and measurement configuration.

As mentioned before, a model for PFXP evaluation is required to calculate areal density. In previous works PFXP was extrapolated from experimental data [4, 7] or from Monte Carlo simulations [9]. Later, many models were proposed for PFXP approximation as a function of experimental conditions and sample properties [10]. In the 1980s Pouchou and Pichoir proposed two of the most popular methods: the PAP (Pouchou and Pichoir) model, in which the distribution function is approximated by two smoothly joined parabolas [11], and the XPP (extended Pouchou–Pichoir) model, which is based on an exponential approximation of the PFXP and allows us to describe an experimental configuration with obliquely incident electrons [12]. In 1981, Packwood and Brown proposed the so-called modified surface-centered Gaussian (MSG) model [13], which is theoretically founded on the hypothesis that electrons move isotropically in the sample. In this model, the persistence of a directional electron propagation near the surface and the consequent deviation from a totally random walk are taken into account, introducing an exponential term which vanishes rapidly with depth. This model was employed by Stenberg and Boman [14], with slightly different parameter definitions, to assess the thickness of amorphous carbon films. In this work, the Rembach–Karduck version of the MSG model, known as the RE method [15], has been considered. The RE method generalizes the MSG model to ultra-soft x-rays emitted by low atomic number materials. The form of the distribution function employed for data analysis is5
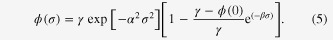
This equation contains four shape-parameters: *α*, whose inverse describes the width of the Gaussian function, is correlated with the penetration depth of incident electrons; *β* takes into account the deviation from a pure Gaussian function in the surface region; *γ* represents the amplitude of the Gaussian function; and 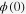 is the surface ionization, the distribution value at the surface. For the definitions of these parameters in the RE model, refer to [10].

The PFXP models described so far are valid for homogeneous samples, while for multilayer samples the electron propagation and x-ray production are altered by the presence of a coating–substrate interface [16]. Thus, in principle, a modified PFXP function should be considered to take into account this effect, but, as far as we know, no analytical model is reported in the literature. As a consequence, x-ray generation distribution functions for both coating and substrate are calculated as the PFXP of a homogeneous sample with the same composition as the layer under analysis. Since the function does not show a strong dependence on the atomic number, this working assumption does not introduce a significant error if the difference between coating and substrate atomic numbers is below 5 [7]. For higher differences, the distortion due to the presence of the coating–substrate interface could introduce an error in areal density measurement.

Moreover, in general, PFXP models should consider the emission of x-rays due to fast secondary electrons (FSE) and fluorescence. In multilayer samples, these effects can be due to the composition of the layer under analysis (matrix effects) but also to the composition of the other layer. The Rehbach–Karduck model adopted in this work only takes into account the FSE matrix effect, whose contribution can be as high as 15% for low-energy x-rays if the initial electron energy is much higher than the absorption edge of the peak under analysis [17]. However, the fluorescence and secondary emission effects due to the sample multilayer structure are not considered in the model.

In addition to the theoretical formulation described so far, a few practical aspects concerning the experimental setup must be taken into account to achieve reliable areal density measurements.

A noteworthy issue is related to the method selection. The coating method and the substrate method are completely equivalent according to the theory, even though their equivalence still has to be experimentally proved. However, in a given experimental configuration one method could be more convenient or more reliable than the other for merely practical reasons. Thus, the availability of two methods is a resource which can be exploited to overcome practical difficulties related to specific experimental configurations. In a few cases, the choice of the method is determined by the specific properties of the sample’s x-ray spectrum. For example, the deconvolution of overlapped x-ray peaks is a time-consuming process that could reduce the technique’s reliability. Thus, the selection of non-resolved peaks should be avoided. Moreover, the choice of particular elements can present critical aspects. For instance, the extremely low energy of carbon K*α* peak (277 eV) could limit the maximum detectable areal density, and the low x-ray production cross-section in the carbon reduces the signal-to-noise intensity ratio. Finally, in the case of multi-elemental coatings, the substrate method can be chosen to remove the issue related to the selection of an appropriate reference standard. In principle, it is not required for the reference standard to have the same composition as the sample coating or substrate. The only requirement is that the emitting element must not be present in both layers. Nevertheless, adopting a standard with the same composition as the material under analysis should reduce the error due to modelling approximations. However, for multi-elemental coatings, it is usually difficult to produce a reference standard with the very same composition as the coating; thus the substrate method should be preferred.

Another issue is related to the selection of an appropriate electron accelerating voltage, which is a crucial issue for areal density measurement, since this parameter determines the methods’ reliability and applicability range. A rough criterion for voltage selection can be deduced from the requirement that the electron initial energy should guarantee a significant energy loss both in the sample coating and in the substrate. Thus, electron energy must be in a range that allows us to probe the sample substrate but in which the effect of the coating on electron propagation is not negligible. Moreover, electron energy cannot be lower than the absorption edge of the emitting element. As a consequence, the lower detection limit of the technique is given by the minimum areal density required to absorb a significant fraction of electron energy for beams with initial energy slightly above the absorption edge. On the other hand, the maximum detectable areal density is lower than the electron penetration range at the maximum available accelerating voltage. In addition, the attenuation of x-rays in the sample can reduce significantly the detected spectral intensity and, as a consequence, the higher detection limit of the technique.

In section 4, the application of EDS-based areal density evaluation methods to coating density measurement will be shown for a number of experimental conditions. Due to its importance for the technique’s reliability, the issue of appropriate electron accelerating voltage selection will be addressed. Moreover, particular attention will be devoted to the choice of the measurement method, and comparisons of the results achieved exploiting the two approaches will be shown whenever possible.

## 3. Experimental methods

Thin films exploited to study the application of EDS-based methods to density evaluation were produced by physical vapour deposition (PVD) techniques. Compact films were produced by thermal evaporation with a base pressure of 5 × 10^−3^ Pa. PLD [18] was employed to grow nanostructured films with density ranging from the solid density to a few 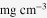.

Reference areal density measurements were carried on for most films using QCMs provided by Inficon: reference coatings were grown on the quartz crystal resonator in the same conditions as the films under analysis.

A Zeiss Supra 40 field-emission SEM was employed to get cross-sectional images for film thickness evaluation (with an accelerating voltage of 5 kV and working distance around 4 mm) and to accelerate electrons for EDS experiments. The latter were carried out with accelerating voltage in the range of 5–30 kV, depending on the expected areal density of the film under analysis. A Si(Li) detector was employed for x-ray spectra collection. System calibration was carried out using Si 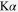 for accelerating voltages below 12 keV and Co 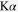 for electron energies above 12 keV. At least three and five spectra were recorded for each reference standard and for each sample, respectively. The acquisition times ranged between 60–120 s, depending on the signal intensity. Peaks considered for each analysed element are reported in table 1.

**Table 1.** X-ray peaks considered for EDS analysis.

Element	Peak	Energy (keV)
C	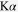	0.277
Al	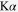	1.49
Si	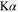	1.74
Rh	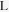 series	2.69
Ag	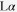	2.98
W	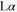	8.37
Au	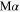	2.12

## 4. Results and discussion

In this section we discuss the results of an extensive experimental campaign intended to apply the methods described in section 2 to evaluate the density of nanostructured thin films once their thickness is known. This section is organized in three subsections corresponding to the main objectives of the experiments.

In section 4.1, we discuss the validation of the technique through a comparison with density measurements achieved using the well-established QCM technique in reliable conditions. The stability of the technique with respect to the electron accelerating voltage selection is discussed, and the effect of the atomic number difference between the sample coating and substrate is investigated. Moreover, results achieved using the substrate method and the coating method are compared to address the issue of method choice.

In section 4.2, we demonstrate the possibility of applying the technique for the evaluation of the density of nanostructured films with known thickness in a wide density and morphology range.

In 4.3, the spatial resolution of the technique is exploited to investigate the properties of non-homogeneous films at both macroscopic and microscopic scales.

### 4.1. Validation of the technique

The first experiment aimed at the validation of the technique was performed using compact Ag films (with expected density 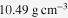) deposited through thermal evaporation on a Au-coated QCM resonator. In this way, EDS and QCM areal density measurements performed on the very same film were compared. The value achieved by the well-established QCM technique, 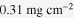, was considered as a reference, since areal density values achieved by QCM are generally very reliable for films grown by thermal evaporation. Ag and Au were chosen as coating and substrate materials, respectively, due to the high atomic number difference between these elements. As discussed in section 2.2, this configuration is not optimal for EDS measurements and allows us to validate the technique and quantify the error in the worst-case scenario.

**Figure 2.**
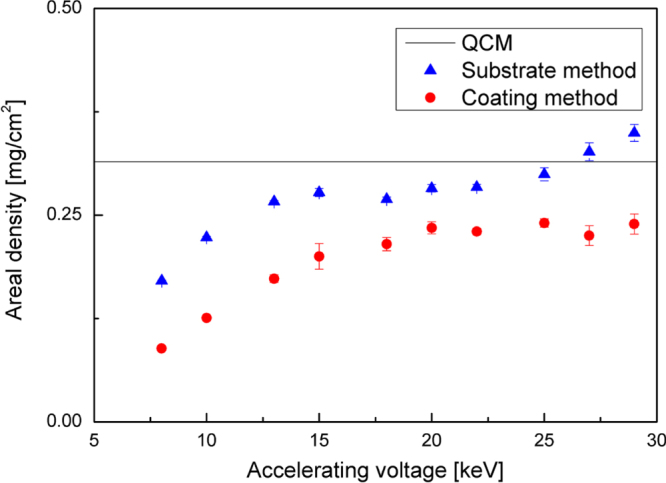
Areal density of a thin Ag film on Au substrate measured as a function of the electron accelerating voltage.

The coating method and the substrate method were employed to characterize the Ag film in a wide electron accelerating voltage range (8–29 kV). From the results reported in figure 2, it is evident that for both methods, an optimal range for the electron accelerating voltage exists in which the areal density values are less affected by voltage variations. For the coating method a voltage higher than 19 kV is required, while the substrate method provides stable results above 13 kV. The maximum acceptable voltage could not be determined, as it is higher than the maximum value achievable with our instruments. The results reported in figure 2 allow us to compare the accuracy of the two methods. In the optimum voltage range, the deviation from QCM-measured areal density is around 10% and 25%, respectively. Thus, in this case, the substrate method is more accurate than the coating method.

**Figure 3.**
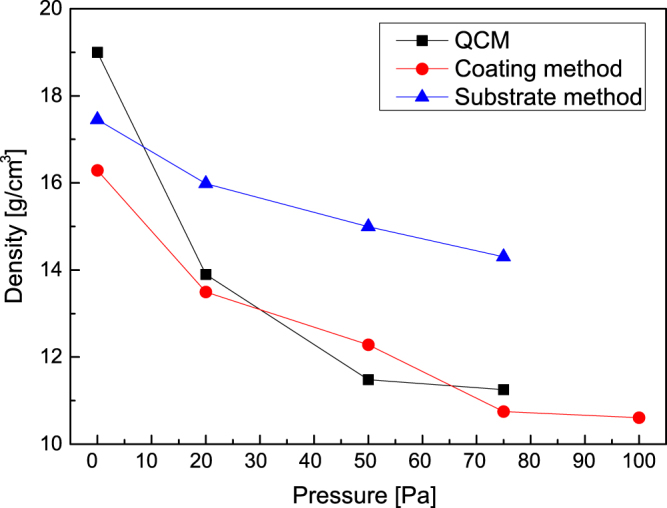
Density of thin tungsten films as a function of gas pressure in the deposition chamber.

The method comparison was further investigated through experiments performed on amorphous-like tungsten films deposited by PLD on Si substrates [19]. In this case, the selection of silicon as substrate material is critical: since the Si 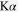 and the W 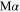 peaks strongly overlap, the accuracy of the substrate method could be non-optimal. In addition, the intensity of the Si K*α* peak can be affected by fluorescence effects due to W L*α* emission in the coating. Results are reported in figure 3. The deviation of measured values from reference density values evaluated by QCM is around 5% for the coating method, much lower than the difference observed for the substrate method, which in some cases is above 30%. Thus, only the coating method results for the W 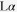 peak will be discussed in section 4.2.

As discussed in section 2, the atomic number difference between the sample coating and substrate can produce a distortion of the PFXP affecting the measurement results. A proof-of-principle experiment was performed on commercially available Al foils with known density and thickness (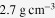 and 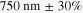) in order to investigate the effect of the substrate atomic number on density values evaluated using the coating method. Two different experimental configurations were considered: measurements were performed on free-standing Al foils and on Al foils arranged on carbon substrates. In both cases, the foil density is systematically underestimated, but the average deviation from the nominal value is around 28% for the free-standing configuration and 20% in the presence of a carbon substrate. This difference can be interpreted, considering that the presence of a carbon substrate enhances the signal from the Al foil. Electrons emerging from the rear side of the Al foil are not necessarily lost by the system but can be scattered back to the foil by C atoms. Thus, the Al PFXP approximation is more reliable in the presence of the C substrate than in the free-standing configuration.

### 4.2. Density measurement of nanostructured films

In this subsection, we illustrate the application of the technique to demonstrate the possibility of evaluating the density of nanostructured films having known thickness. A variety of film morphologies are considered, resulting in films with density ranging from the solid density to a few 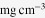. Results reported in this section refer to films grown by PLD. Density values achieved by QCM are considered as a reference, even though densities achieved in PLD facilities also can be affected by an error for compact films (around 5–10% for our experimental setting) due to the difficulty of placing the sensor in the very same position as the substrate.

The reliability of EDS for density measurement of nanostructured films in a wide density range was tested, exploiting amorphous-like tungsten films on Si substrates [19].

In figure 3, density values evaluated using both the coating method and QCM are plotted as a function of the gas pressure in the deposition chamber during the film growth process. The results show a very good agreement between coating method and QCM, except for the sample deposited in vacuum. The reason for this behaviour could be the low thickness of W coatings deposited in vacuum (less than 100 nm), whose evaluation through SEM cross-section analysis might be affected by an error of around 10%.

**Figure 4.**
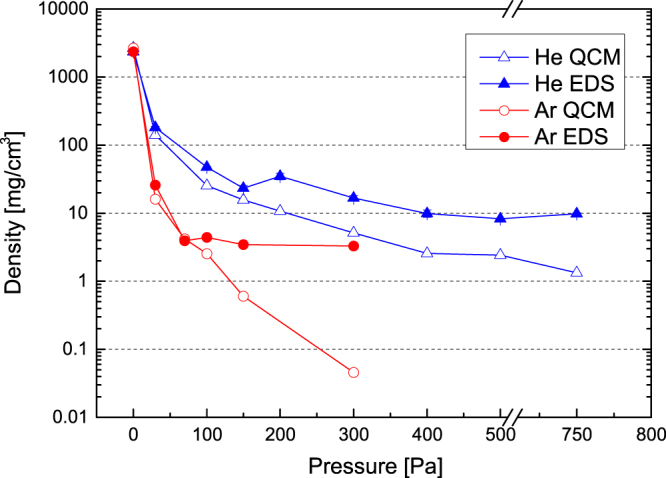
Density of carbon foams as a function of gas pressure in deposition chamber.

A second experiment aimed at testing the application of the technique to nanostructured films was performed, exploiting carbon foam densities down to a few 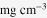 [3]. In this case, the substrate method was selected for the reasons discussed in section 2.2. In figure 4, results for both the substrate method and QCM are shown as a function of the gas pressure in the deposition chamber. The agreement between the two methods is satisfactory only for density values above 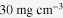. For lower densities, values measured by QCM are unrealistically low, as QCM undergoes a sensitivity loss due to the very porous foam structure, which results in a decoupling between the film and the quartz crystal resonator [20]. On the contrary, the substrate method shows a more plausible density saturation for increasing gas pressure, which is typical of this kind of deposition process. Thus, EDS-based methods can be applied for films produced by PVD for densities down to a few 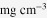, also in a density range in which QCM is not reliable.

**Figure 5.**
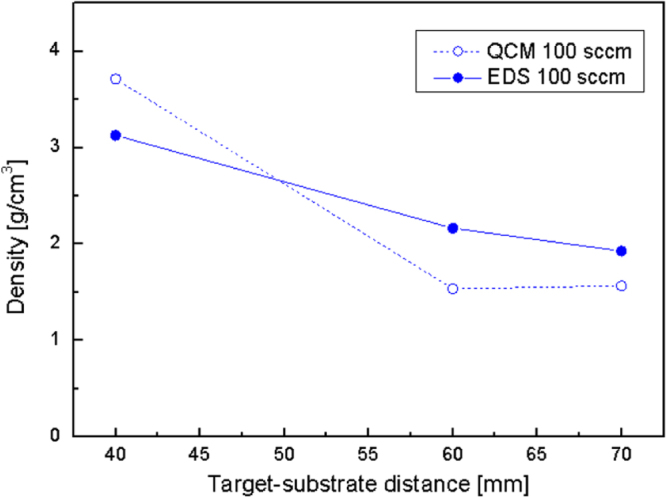
Density of Al-doped ZnO films as a function of the target-to-substrate distance.

The possibility of employing EDS for density evaluation in the case of multi-elemental coatings was tested, exploiting aluminium-doped zinc oxide nanostructured films [21]. In this case, the substrate method was chosen due to the unavailability of reference standards with the same composition as the films under analysis. In figure 5, results achieved using the substrate method and QCM are shown as a function of the target-to-substrate distance. In this case, a strong uncertainty affects density values achieved by QCM, because the deposition configurations adopted for film growth and for QCM measurements were not equivalent. However, density trends predicted by QCM are confirmed by the substrate method. Thus, as stated in [21], a decreasing trend in the film density with the target-to-substrate distance is observed.

### 4.3. Spatially resolved density measurements

One of the most interesting characteristics of EDS is its spatial resolution, which can be exploited for the characterization of non-uniform films at a macroscopic scale, i.e., for density profile evaluation, and at a microscopic scale, for the evaluation of the characteristic inhomogeneity length of a material.

**Figure 6.**
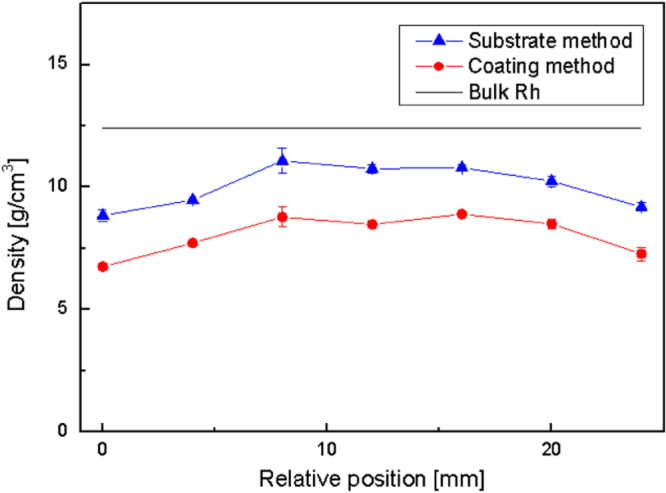
Density profile of a Rh nanocrystalline film.

The density profiles of Rh nanocrystalline coatings were measured along a cross-section exploiting both the coating method and the substrate method. Density was calculated from areal density, and thickness values were measured in the very same points. The results reported in figure 6 refer to a Rh film with a non-uniform thickness profile and thickness ranging from about 70 to 135 nm. A non-uniform density profile is evident for both methods. Film density is approximately constant in the central region of the sample, while a 15% decrement is observed in the peripheral deposit region, where the film thickness is lower than 70 nm. As the film was deposited in vacuum [22], the coating density was expected to be very close to the bulk density value for Rh (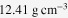). In the central region of the coated surface, film density measured by the substrate method is around 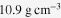, while the coating method gives a density of about 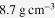. In both cases density is underestimated with respect to the expected value. Since the results achieved by the substrate method are closer to the expected film density, the substrate method can be considered more reliable than the coating method in this case.

**Figure 7.**
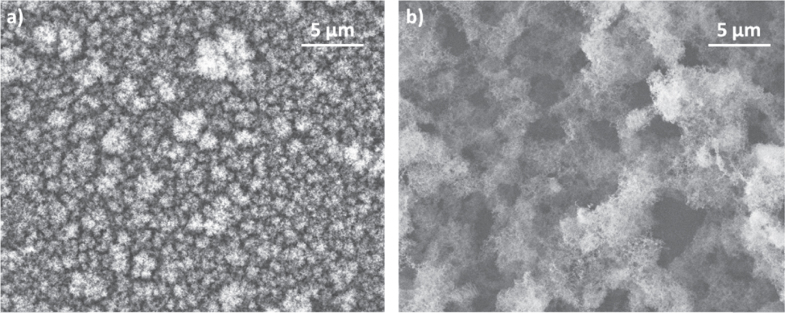
SEM images of carbon films deposited in Ar at 30 Pa (a) and 300 Pa (b).

**Figure 8.**
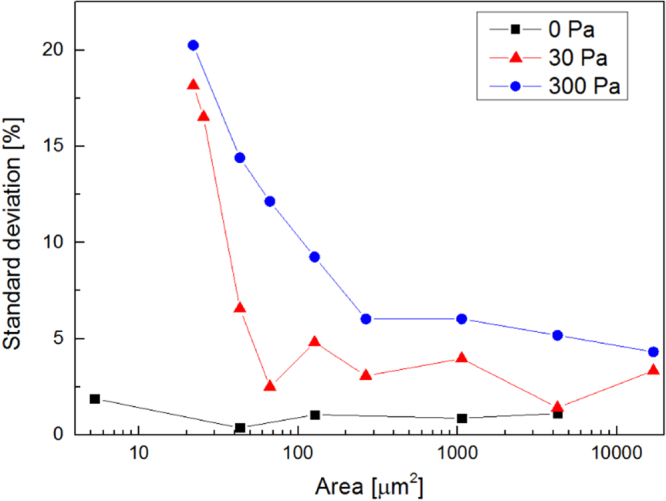
Standard deviation of areal density measurements for carbon films deposited in vacuum and in argon with 30 Pa and 300 Pa.

The application of EDS to the analysis of mesoscale inhomogeneity was investigated, exploiting carbon films [3]. In general, EDS scans performed on a wide film area result in a relatively low areal density standard deviation, since measured areal density values are averaged on a large surface. As the sampled region is reduced, standard deviation increases if the film presents inhomogeneities with a length scale comparable with the diameter of the sampled region. Thus, the inhomogeneity length scale can be estimated as the sampled area for which areal density standard deviation suddenly starts increasing. This approach was developed to introduce a quantitative criterion to compare films with qualitatively similar mesoscale structures. To this aim, we analysed carbon films produced by PLD with different inhomogeneity length scales: a compact coating produced in vacuum and two foams produced using argon as buffer gas with pressures around 30 Pa and 300 Pa. These films have different mesoscale morphologies (see figure 7) and, as a consequence, different densities (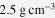, 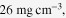 and 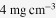, respectively). Results are illustrated in figure 8. The sampled surface area ranges from 10 to 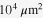. The areal density standard deviation for the compact coating is stable even for high magnifications. For carbon foam layers produced in argon at 30 and 300 Pa, a sudden increase is observed as the sampled area decreases below 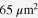 and 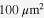, respectively. Thus, the inhomogeneity length scales can be estimated as 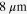 and 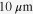. These values confirm the morphological difference evident from SEM images and provide a quantitative criterion to compare the inhomogeneity length scale of films with similar morphology.

### 4.4. Discussion

The experimental results reported in this paper allow us to draw general conclusions regarding the application of EDS to the evaluation of nanostructured thin film density and provide useful indications regarding its practical applications for density measurements.

The first observation is related to the choice of the EDS-based method. Although the technique accuracy is strongly dependent on the specific experimental configuration and, in particular, on the substrate–coating combination, in general the substrate method can be considered more reliable than the coating method. For the substrate method, the error with respect to values measured by QCM is around 10–15%, but it can reach values up to 30% if the substrate–coating combination is particularly unfavourable, for example, because of peak overlapping. Nevertheless, in a few cases (i.e., W films on Si substrates) the coating method allows us to achieve very reliable measurements with an extremely low deviation from nominal density values.

The strong dependence of the technique accuracy on the substrate–coating combination has been considered in subsection 4.1. Apparently, this factor is the main error source in density measurements performed by EDS. In principle, the precision could be enhanced by developing an appropriate model for PFXP in multilayer samples, taking into account also fluorescence and secondary emission effects. However, the accuracy of both the coating method and the substrate method can be enhanced by adopting suitable conditions, namely selecting substrates with an atomic number similar to the coating atomic number. Moreover, the effect of the characteristic properties of the model used to evaluate the PFXP—in this case the MSG model—on the accuracy of the two methods should be considered.

Also, the electron accelerating voltage selection plays an important role in areal density evaluation. This issue has been extensively discussed in section 2. Moreover, the stability of the technique with respect to accelerating voltage variations has been studied from 5–30 keV for Ag films deposited on Au substrates. An empirical method to verify that the selected voltage is included in the stability voltage range consists in checking the measure and repeating it with slightly higher and lower acceleration voltages for a test film. If the measured density does not change, the accelerating voltage falls in the optimum range.

## 5. Conclusions

In conclusion, we discussed the quantitative development and test of two methods based on EDS and cross-sectional SEM images for thin film density measurement. We demonstrated the applicability of these methods to a number of different experimental conditions: thin films with various compositions, different coating–substrate combinations, various mesoscale morphologies, and with densities in an extremely wide range (few 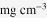
–20 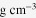). Moreover, a novel application of EDS to the analysis of coating inhomogeneity at the macroscopic and microscopic scales has been shown.

Although the results can be affected by an error up to 30% in a few unfavourable configurations, in general this technique guarantees a reliable, fast, simple, and cheap measurement process to evaluate the density of nanostructured thin films in a wide range of morphologies and compositions, exploiting the common integrated EDS–SEM equipment present in most material science laboratories.
